# Personality Traits in Marathon Runners and Sedentary Controls With MMPI-2-RF

**DOI:** 10.3389/fpsyg.2020.00886

**Published:** 2020-05-08

**Authors:** Astrid Roeh, Rolf R. Engel, Moritz Lembeck, Benjamin Pross, Irina Papazova, Julia Schoenfeld, Martin Halle, Peter Falkai, Johannes Scherr, Alkomiet Hasan

**Affiliations:** ^1^Department of Psychiatry and Psychotherapy, University Hospital, LMU Munich, Munich, Germany; ^2^Department of Prevention and Sports Medicine, Klinikum Rechts der Isar, Technischen Universität München, Munich, Germany; ^3^University Center for Preventive and Sports Medicine, Balgrist University Hospital, University of Zurich, Zurich, Switzerland; ^4^Department of Psychiatry, Psychotherapy and Psychosomatics of the University Augsburg, Bezirkskrankenhaus Augsburg, University of Augsburg, Augsburg, Germany

**Keywords:** personality, exercise, endurance training, marathon, MMPI

## Abstract

**Background:**

Endurance exercise in general and marathon running in particular have become increasingly popular over the past decades. Recent investigations about personality structures in this cohort and comparisons to non-active cohorts are lacking.

**Methods:**

In the ReCaP study (Running effects on Cognition and Plasticity), a total of 100 marathon runners and 46 sedentary controls were recruited. After elimination of Minnesota Multiphasic Personality Inventory 2 Restructured Form (MMPI-2-RF) profiles with insufficient validity, 79 marathon runners (MA) and 27 sedentary controls (SC) remained for final analyses. Depressive symptoms were evaluated with Beck Depression Inventory (BDI) and Hamilton Depression Scale (HAMD).

**Results:**

Marathon runners had lower scores in scales measuring somatic and cognitive complaints, stress, demoralization, hopelessness and distrust. Within the marathon group, committed runners exhibited hypomanic traits compared to regular runners.

**Discussion and Conclusion:**

Personality differences could be summarized as (sub-)depressive personality traits in SC compared to MA rather than typical (sub-) depressive symptoms in the meaning of depressive disorders. Future studies should further evaluate cause and consequence of endurance training and hypomanic or euthymic symptoms, as a two-way interaction exists.

**Trial Registration:**

http://apps.who.int/trialsearch/Trial2.aspx?TrialID=DRKS00012496.

## Introduction

Endurance training and competitions like marathon running have become increasingly popular over the past decades. The beneficial effects of regular exercise training on the cardiovascular system are well-established (Praet and van Loon, 2009; Dimeo et al., 2012). In somatic and neuropsychiatric diseases, regular physical exercise has been shown to prevent depressive symptoms (Schuch et al., 2018; Roeh et al., 2019b) and improve cognitive symptoms (Buchman et al., 2012).

Regarding the psychological aspects of physical activity and especially of marathon running, consistent findings are lacking. Prior research aimed at identifying specific personality traits of committed runners. Different personality questionnaires and definitions were used, the most popular representing personality traits according to the Big Five Model or modifications (Goldberg, 1993). Committed marathon runners scored similar to the general population on the 16 personality factor (PF) questionnaire (McLeavey et al., 1984). It was assumed that (ultra-) marathon runners participate in the sport for several reasons, pointing toward specific personality traits (e.g., openness) in this cohort: a sense of achievement, a challenge, the opportunity to extend current capabilities, and the opportunity to socialize with other runners and meet people (Hashimoto et al., 2006). Committed athletes (11 h or more training per week) seem to be more extraverted than average sportsmen (less than 4 h training per week) (Egloff and Jan Gruhn, 1996).

Similar, but potentially even more pronounced personality traits regarding running-specific aspects, should be displayed in marathon and ultra-marathon runners. A systematic review of comparisons of ultra-marathon runners with the general population using various different questionnaires [e.g., NEO-Five Factor Inventory (NEO-FFI), Sensation Seeking Scale (SSS), Temperament and Character Inventory (TCI), Ten Item Personality Inventory (TIPI), Minnesota Multiphasic Personality Inventory (MMPI), Flow State Scale (FSS), State Trait Anxiety Inventory (STAI)] found conflicting evidence: in two studies the athletes were described as more self-motivated, more extraverted, open and experience-seeking and less disinhibited (*N* = 44 and 66 participants) (Rauch et al., 1988; Hughes et al., 2003); but other included studies found the ultra-marathon runners (*N* = 52 and 22 participants) to be more introverted and more self-transcendent and less cooperative and reward-dependent (Hashimoto et al., 2006; Freund et al., 2013; Roebuck et al., 2018). Another study showed no differences in ultra-marathon runners (*N* = 50) compared to non-runners using the Self-Motivation Inventory (SMI) and four subscales from the Philosophies of Human Nature (McCutcheon and Yoakum, 1983). In summary, consistent findings of personality traits in the (ultra-) marathon population are still lacking.

A recent narrative review on personality aspects (measured with, e.g., Cattell 16 Personality Factor Questionnaire, Karolinska Scales of Personality (KSP), adjective check list, The Self-Motivation Inventory (SMI), the Philosophies of Human Nature) of marathon runners described this population as having low scores in tension, depression, anger, fatigue and confusion, but high scores in vigor (Morgan and Costill, 1996; Nikolaidis et al., 2018). Many of the included studies and analyses were performed in the 1980s, a time where marathon running was not a trend sport. Moreover, the composition of the running cohort has changed over years from mainly young male to a mixed cohort with more women and aged runners today. This development results in a need for more comprehensive examinations of personality aspects in this newly reassembled cohort (Nikolaidis et al., 2018).

To the best of our knowledge, this is the first study to evaluate the personality traits of marathon runners with different subgroups (e.g., committed runners, sex, age) using the newly developed Minnesota Multiphasic Personality Inventory 2 Restructured Form (MMPI-2-RF) and compare them to age- and gender matched sedentary controls in an exploratory analysis. The revised form offers evaluations with regard to dimensional models of psychopathology and therefore improves categorization of symptoms.

With our design of exercise-specific subgroups in the marathon cohort and the sedentary control group we expect to be able to identify more specifically the personality traits in relation to daily activity. We hypothesized that persons who did marathon running or intend to do so will experience less distress and somatic complaints compared to the non-active group and that these traits will be more pronounced in the most committed runners.

## Design and Methods

### Subjects

The study population was part of the ReCaP trial (“Running effects on Cognition and Plasticity,” a longitudinal observational study of marathon runners who were registered for the Munich Marathon that took place on October 8th, 2017). The detailed study protocol was previously published (Roeh et al., 2019a).

A total of 146 participants (100 marathon group, 46 sedentary control group) were included in the trial. 116 participants (83 marathon group and 33 sedentary control group) filled out the MMPI-2-RF with less than 16 missing items.

Inclusion criteria for the marathon group (MA) contained an age range between 18 and 60 years, successful registration for Munich Marathon 2017, participants must have completed at least one half-marathon prior to the recent event, sufficient German language skills and written informed consent. Exclusion criteria contained relevant neurological, cardiac or psychiatric diseases, pregnancy and cannabis abuse, BMI > 30 kg/m^2^.

The sedentary control group (SC) was age- and gender matched with identical exclusion criteria. They were not included in case of participation in a marathon or half-marathon in earlier years. A sedentary lifestyle was defined as less than 25 min of physical activity per day (Cabrera de León et al., 2007). The participants of both groups filled out the questionnaires in person.

### Ethics and Registration

The study proceedings agreed with Good Clinical Practice guidelines, the guiding principles of the Declaration of Helsinki 2008, and local laws and regulations. The study protocol had been approved by the ethics committees of both the Ludwig-Maximilians University Munich (approval reference number 17–148) and the Technical University Munich (approval reference number 218/17 S). The study was registered at WHO International Clinical Trials Research platform^1^ (registration number: DRKS00012496). All participants provided written informed consent prior to inclusion in the study.

### Instruments

#### Personality Traits

Baseline values of personality aspects were assessed via the German translation of the Minnesota Multiphasic Personality Inventory 2 Restructured Form (MMPI-2-RF) (Moultrie and Engel, 2017). Table 1 provides an overview of all measured subscales.

**TABLE 1:** MMPI-II RF overview of subscales.

	Validity scales		
VRIN-r	Variable response inconsistency*°		**Internalizing scales**
TRIN-r	True response inconsistency	SUI	Suicidal/Death ideation
F-r	Infrequent responses*°	HLP	Helplessness/Hopelessness*
Fp-r	Infrequent psychopathology responses*	SFD	Self-doubt
Fs	Infrequent somatic responses	NFC	Inefficacy
FBS-r	Symptom validity°	STW	Stress/Worry*
RBS	Response bias scale	AXY	Anxiety
L-r	Uncommon virtues	ANP	Anger proneness
K-r	Adjustment validity	BRF	Behavior-restricting fears
	**Substantive scales**	MSF	Multiple specific fears
HO	**Higher-order scales**		**Externalizing scale**
EID	Emotional/Internalizing dysfunction	JCP	Juvenile conduct problems°
THD	Thought dysfunction*	SUB	Substance abuse
BXD	Behavioral/Externalizing dysfunction	AGG	Aggression°
RC	**Restructured clinical scales**	ACT	Activation
RCd	Demoralization*		**Interpersonal scales**
RC1	Somatic complaints*	FML	Family problem
RC2	Low positive emotions	IPP	Interpersonal passivity
RC3	Cynicism°	SAV	Social avoidance°
RC4	Antisocial behavior	SHY	Shyness
RC6	Ideas of persecution*	DSF	Disaffiliativeness°
RC7	Dysfunctional negative emotions		**Interest scale**
RC8	Aberrant experiences*	AES	Aesthetic-literary interests
RC9	Hypomanic activation°	MEC	Mechanical-physical interests
SP	**Specific problem scales**	PSY-5	**Personality psychopathology five scale**
	**Somatic/Cognitive scales**	AGGR-r	Aggressiveness-revise
MLS	Malaise	PSYC-r	Psychoticism-revised
GIC	Gastrointestinal complaints°	DISC-r	Disconstrained-revised°
HPC	Head pain complaints*	NEGE-r	Negative emotionality/Neuroticism-revised
NUC	Neurological complaints	INTR-r	Introversion/Low positive emotionality-revised
COG	Cognitive complaints*		

*Significant difference between marathon runners and non-active group; °significant differences between MMPI median-split marathon runners.

#### Physical Activity Assessment

We used the International Physical Activity Questionnaire (IPAQ) to present detailed information about daily activity including physical exercise as well as physical activity in daily routines (e.g., usage of the bicycle for work) (Craig et al., 2003). The findings of the questionnaire show high correlation with activity measured with accelerometers (Mäder et al., 2006).

In the marathon runners, we further assessed the physical activity with training protocols about their training history in the 12 weeks prior to the marathon (including mean training kilometers per week and the mean weekly training duration). We collected information about their prior participations in marathon events with questionnaires. Moreover, we measured the endurance capacity with a spiroergometry (performed at visits −1 and 0). The main outcome parameter for our analyses was V0_2_max [milliliters of oxygen used in 1 min per kilogram of body weight (mL/kg/min)].

#### Mood Questionnaires

Two rating scales for depressive symptoms were included for the here presented analyses. The self-rating scale Beck Depression Inventory (BDI) (Beck et al., 1961) and the observer-rating scale Hamilton Depression Scale (HAMD) (Hamilton, 1960).

### Procedures

After comparisons of marathon group versus sedentary control group, we divided the marathon group in two activity-dependent subgroups for further analyses. In literature, no clear definitions of “committed runners” could be identified [one study used less than four and more than 11 weekly training hours (Egloff and Jan Gruhn, 1996)]. Thus, three different exploratory approaches with identical sample sizes were used in our sample: a median-split separation of the marathon population with the International Physical Activity Questionnaire (IPAQ) baseline score (Craig et al., 2003), a median-split separation with the individual marathon finishing time and a median-split separation with the spiroergometry performance expressed as VO_2_max.

Two further comparisons were included in the analysis: we compared male and female runners and younger and older participants (also using a median-split approach as we could not identify an established age separation in literature concerning the study question).

### Statistical Analysis

As all participants were healthy (exclusion criterion of the study: relevant psychiatric disease), the inclusion criteria for final analyses were further restricted with the following validity scales: TRIN-r < 80T (one sedentary control group member (SC) and one marathon member (MA) were excluded); VRIN-r < 80T (one SC and two MA were excluded); F-r < 80T (two SC and one MA were excluded); Fp-r < 80T (5 SC and 3 MA were excluded). Some of these parameters referred to the same participants resulting in an exclusion of 10 participants (6 SC and 4 MA) for final analyses with 106 participants (79 MA, 27 SC) remaining.

All analyses were carried out in SPSS25 (IBM, Armonk, NY, United States), with a significance level of α = 0.05. Differences between groups were assessed using χ^2^-tests (Fisher’s exact test if> 20% of cells had an expected count < 5), two-sided independent *t*-tests and in cases of a violation of the normal distribution (Shapiro–Wilk Test, *p* < 0.05) with Mann–Whitney-*U* Test. For demographic data, BMI was normally distributed and for MMPI data, K_r, RC3, RC9 and AGGR_r were normally distributed (Shapiro–Wilk Test, *p* > 0.05).

Only complete datasets for a given dependent variable were analyzed (please see respective tables for the sample sizes for each variable). The tables show mean values and standard deviation.

## Results

Baseline demographic results of the included participants and their comparison between marathon and sedentary control group are presented in Table 2. They were similar in educational status, age and gender. Significant differences were found in smoking habits and less pronounced in BMI values. The differences in IPAQ scores were expected due to inclusion criteria.

**TABLE 2:** Baseline characteristics of marathon group and sedentary controls, MA: marathon group, SC: Sedentary Controls.

	N (MA/SC)	MA		SC		MA vs. SC		
						χ^2^	*df*	*p*
***Demographics***								
Gender (male : female)	106 (79/27)	65:14		20:7		0.85	1	0.356
Smoking (yes : no)	102 (75/27)	0:75		7:20				< 0.001^*a*^

		**Mean**	**SD**	**Mean**	**SD**	***T***	***df***	***p***

BMI (kg/m^2^)	106 (79/27)	23.39	2.55	24.38	2.56	−1.75	104	0.083

						***U***	***Z***	***p***

Age (years)	106 (79/27)	43.58	9.94	40.81	12.17	931.0	−0.98	0.328
Education (years)	101 (74/27)	15.26	3.97	14.31	3.12	911.0	−0.68	0.498
IPAQ	103 (78/25)	7002.94	6661.98	3482.96	6022.81	340.0	−4.89	< 0.001
BDI	102 (76/26)	2.58	3.97	5.85	5.06	539.5	−3.49	< 0.001
HAMD	104 (77/27)	1.99	3.47	2.37	3.21	964.0	−0.58	0.563

*Analysis after elimination of invalid questionnaires or non-existing MMPI data; *T*-Test with presentation of T, df, p; Mann–Whitney-*U* Test with presentation of U, Z, p; ^*a*^two-tailed Fisher’s exact test.*

### Comparison Between Marathon Group and Sedentary Control Group

The results of both the validity and the clinical subscales of MMPI-2 RF with significant differences between both groups are presented in Table 3.

**TABLE 3:** Demographics of MMPI scales and comparison between MA (marathon group) and SC (sedentary controls).

	N (MA/SC)	MA		SC		MA vs. SC		
*Validity scales*		Mean	SD	Mean	SD	U	Z	p
VRIN	106 (79/27)	45.31	9.38	54.45	10.81	775.5	−2.12	0.034
F	106 (79/27)	46.92	6.36	52.17	9.98	744.0	−2.36	0.018
Fp	106 (79/27)	48.58	7.18	53.33	10.20	786.5	−2.06	0.039
***Higher-Order Scales***								
THD	106 (79/27)	47.88	7.60	53.61	11.56	727.0	−2.48	0.013
***Restructured Clinical Scales***								
RCd	106 (79/27)	48.34	8.34	54.81	13.73	790.5	−2.02	0.043
RC1	106 (79/27)	44.48	4.40	47.89	6.58	759.5	−2.25	0.024
RC6	106 (79/27)	47.15	8.10	52.69	12.08	739.5	−2.40	0.016
RC8	106 (79/27)	48.55	7.88	53.93	10.16	732.0	−2.46	0.014
***Somatic/Cognitive Scales***								
HPC	106 (79/27)	46.15	5.93	50.35	7.88	755.5	−2.35	0.018
COG	106 (79/27)	50.41	9.04	56.44	12.83	780.0	−2.12	0.034
***Internalizing Scales***								
HLP	106 (79/27)	43.93	8.31	48.38	10.18	789.5	−2.07	0.038
STW	106 (79/27)	49.42	10.52	54.83	11.68	780.0	−2.10	0.036

*VRIN, variable response inconsistency; F, infrequent responses; Fp, infrequent psychopathology responses; RCd, Demoralization; RC1, somatic complaints; RC6, ideas of persecution; RC8, aberrant experiences; THD, thought dysfunction; HPC, head pain complaints; COG, cognitive complaints; HLP, helplessness/hopelessness; STW, stress/worry; Mann–Whitney-*U* Test with presentation of U, Z, p.*

Differences between both groups regarding BDI and HAMD scores are shown in Table 2, they only differed in BDI values (self-rating), not in HAMD values (observer-rating). All values were within normal ranges (cut-off for mild depressive disorder is nine points in BDI).

### Comparison Within the Marathon Group

Of the 100 included participants, 71 finished the marathon with a median time of 231.84 min (range 167.65 – 346.82). Median instead of mean was presented because of median-split comparison. Of these, 60 provided sufficient MMPI-2 RF data. Of the 100 participants, 92 participants completed the baseline spiroergometry and 78 of this cohort provided sufficient MMPI-2 RF data. Median maximal oxygen uptake was 47.89 ml/kg/min (range 25.4 – 63.5). IPAQ baseline results of 92 participants resulted in a median of 4986.75 MET-minutes per week (Metabolic Equivalent) (range 910.00 – 42360.0). 78 participants remained after application of the above described validity and quality parameters. Of the 79 participants with sufficient MMPI 2 RF data, 77 provided more detailed information about their participation in prior marathon events: 59/77 had already participated in prior marathon events before Munich Marathon 2017 (all participants had prior experience in half marathons, see inclusion criteria). Mean training hours exclusively for running was 4.77 ± 2.27 per week (*N* = 64) with a mean weekly running frequency of 3.66 ± 1.33 (*N* = 68). Mean weekly training kilometers were 45.36 ± 21.20 (*N* = 69).

Results of the comparisons within the marathon population using the marathon finishing time or maximal oxygen uptake in the baseline spiroergometry showed no significant differences between both groups regarding personality aspects. Table 4 displays the personality parameters with significant differences between high- and low performers using the median-split comparison of the baseline IPAQ score. The respective scales were highlighted in Table 1 for better comparability. HAMD and BDI scores showed no significant differences in both median-split IPAQ and finishing time scores.

**TABLE 4:** Demographics of MMPI scales and comparison between IPAQ HP (high performer) and IPAQ LP (low performer).

	N (HP/LP)	IPAQ HP		IPAQ LP		IPAQ HP vs LP		
*Restructured Clinical Scales*		*Mean*	*SD*	*Mean*	*SD*	*T*	*df*	*p*

RC3	78 (39/39)	42.39	8.89	36.66	10.74	2.564	76	0.012
RC9	78 (39/39)	49.28	9.17	44.59	9.02	2.278	76	0.026

*Validity scales*						*U*	*Z*	*p*
VRIN_r	78 (39/39)	51.78	10.01	47.24	8.29	544.5	−2.17	0.029
F	78 (39/39)	48.81	7.31	45.21	4.64	515.5	−2.48	0.013
FBS	78 (39/39)	47.24	5.09	50.12	6.00	537.0	−2.25	0.024
***Somatic Complaints***								
GIC	78 (39/39)	45.61	3.40	48.07	7.18	507.0	−3.26	0.001
***Externalizing Scale***								
JCP	78 (39/39)	49.91	10.11	45.61	7.43	558.0	−2.09	0.036
AGG	78 (39/39)	48.43	8.40	45.37	8.13	555.0	−2.09	0.036
***Interpersonal Scale***								
DSF	78 (39/39)	52.28	11.75	46.86	8.14	534.5	−2.44	0.015
SAV	78 (39/39)	52.12	9.25	48.47	7.40	559.5	−2.02	0.043
***Personality Psychopathology Five Scale***								
DISC-r	78 (39/39)	52.33	9.73	47.90	6.61	534.5	−2.27	0.023

*VRIN, variable response inconsistency; F, infrequent responses; FBS, symptom validity; GIC, gastrointestinal complaints; RC3, cynicism; RC9, hypomanic activation; JVP, juvenile conduct problems; AGG, aggression; DSF, disaffiliativeness; SAV, social avoidance; DICS-r, disconstrained-revised; *T*-Test with presentation of *T, df, p*; Mann–Whitney-*U* Test with presentation of *U, Z, p*.*

To further investigate the effect of gender and age, we performed subsequent analyses. First, we compared the marathon group with regard to differences between male and female runners. Male and female runners (m:f = 65:14) showed differences in age (m:f = 45.14 ± 9.49 : 36.36 ± 9.0, *U* = 223.5, *Z* = −2.98, *p* = 0.002), BMI (m:f = 23.70 ± 2.53 : 21.96 ± 2.20, *T* = −2.38, *df* = 77, *p* = 0.016) and BDI (m:f = 2.0 ± 3.07 : 5.38 ± 3.15, *U* = 128.5, *Z* = −3.96, *p* < 0.001). Please see Table 5 for MMPI results of MMPI scales with male/female.

**TABLE 5:** Demographics of MMPI scales and comparison between males (m) and females (f) within the marathon group.

	N (m/f)	Male		Female		m vs. f		
*Validity scales*		*Mean*	*SD*	*Mean*	*SD*	*U*	*Z*	*p*
VRIN	79 (65/14)	58.14	9.01	55.41	9.02	253.5	−2.61	0.008
F	79 (65/14)	46.13	5.65	50.60	8.24	234.5	−2.87	0.003
L	79 (65/14)	47.96	9.90	43.15	7.01	271.0	−2.38	0.016
***Somatic Complaints***								
GIC	79 (65/14)	46.74	5.17	47.20	7.86	129.0	−5.40	< 0.001
***Restructured Clinical Scales***								
RCd	79 (65/14)	47.37	8.16	52.85	7.94	211.5	−3.16	0.001
RC6	79 (65/14)	46.30	7.78	51.10	8.68	247.0	−2.72	0.006
***Internalizing Scale***								
NFC	79 (65/14)	45.90	8.39	54.29	9.74	245.5	−2.73	0.006
***Externalizing Scale***								
SUB	79 (65/14)	51.81	9.64	60.23	12.32	254.5	−2.63	0.008
***Interpersonal Scale***								
DSF	79 (65/14)	48.68	10.24	53.19	10.55	216.5	−3.31	0.001
***Interest Scales***								
MEC	79 (65/14)	47.24	8.53	54.16	7.95	240.0	−2.79	0.004
***Personality Psychopathology Five Scale***								
NEGE	79 (65/14)	54.86	9.06	54.01	8.93	227.0	−2.94	0.003

*VRIN, variable response inconsistency; F, infrequent responses; L, uncommon virtues; GIC, gastrointestinal complaints; RCd, demoralization; RC6, ideas of persecution; NFC, inefficacy; SUB, substance abuse; DSF, disaffiliativeness; MEC, mechanical-physical interests; Nege, negative emotionality/neuroticism; Mann–Whitney-*U* Test with presentation of *U, Z, p*.*

Next, we divided our marathon cohort with regard to age using a median-split. Median age of the marathon cohort was 44.0 years (range 23 – 60 years), the younger group was composed of participants aged 23 – 44 years (mean 35.0 ± 5.72 years) and the older group had an age range of 44.1 – 60 years (mean 52.38 ± 3.49 years). Younger and older participants (40:39) differed significantly with regard to the gender distribution [younger group f:m = 11:29 and older group f:m = 3:36, *df* = 1, *p* = 0.037 (two-tailed Fisher’s exact test)], but showed no significant differences in the other parameters (BMI, BDI, IPAQ, HAMD, education). Significant differences of MMPI parameters of this comparison are presented in Table 6.

**TABLE 6:** Demographics of MMPI scales and comparison between younger (y, 23 – 44 years) and older marathon participants (o, 44.1 – 60 years).

	N (y/o)	Young (y)		Old (o)		y vs. o		
		*Mean*	*SD*	*Mean*	*SD*	*U*	*Z*	*p*
***Validity scales***								
VRIN	79 (40/39)	52.15	10.53	46.63	7.15	535.5	−2.41	0.015
***Substantive Scales***								
THD	79 (40/39)	49.95	8.14	45.75	6.44	530.0	−2.48	0.013
BXD	79 (40/39)	50.48	8.59	46.31	8.21	540.0	−2.36	0.018
***Restructured Clinical Scales***								
RCd	79 (40/39)	50.55	9.08	46.09	6.91	514.5	−2.63	0.008
RC8	79 (40/39)	50.71	8.94	46.34	5.97	558.5	−2.21	0.027
RC9	79 (40/39)	49.68	9.21	43.86	8.65	493.0	−2.82	0.004
***Somatic/Cognitive Scale***								
COG	79 (40/39)	53.29	9.58	47.45	7.46	505.0	−2.76	0.005
**Internalizing Scale**								
SFD	79 (40/39)	53.16	11.88	47.43	8.35	593.5	−2.01	0.044
AXY	79 (40/39)	52.41	11.62	45.70	5.25	577.0	−2.29	0.022
MSF	79 (40/39)	47.15	7.83	43.76	7.72	537.5	−2.42	0.015
***Interpersonal Scale***								
FML	79 (40/39)	52.78	10.04	47.97	8.50	573.5	−2.05	0.040
DSF	79 (40/39)	52.07	10.91	46.82	9.18	595.5	−3.02	0.002
***Interest Scales***								
MEC	79 (40/39)	50.66	9.21	46.21	7.84	558.5	−2.20	0.028
***Personality Psychopathology Five Scale***								
DISC	79 (40/39)	52.15	8.88	47.91	9.33	551.5	−2.25	0.024
INTR	79 (40/39)	49.97	8.31	52.83	7.94	566.5	−2.11	0.035

*VRIN, variable response inconsistency; THD, thought dysfunction; BXD, behavioral/externalizing dysfunction; RCd, demoralization; RC8, aberrant experiences; RC9, hypomanic activation; COG, cognitive complaints; SFD, self-doubt; AXY, anxiety; FML, family problem; DSF, disaffiliativeness; MEC, mechanical-physical interests; DISC, disconstrained; INTR, introversion/low positive emotionality; Mann–Whitney-*U* Test with presentation of *U, Z, p*.*

## Discussion

In our study, we compared marathon runners with an age- and gender-matched non-active control group in regard to personality aspects using the newly developed MMPI 2 RF (German translation).

We identified three superordinate groups of clinical subscales with differences between marathon runners and sedentary controls: Marathon runners seem to experience less *physical and cognitive complaints*, as their lower results in the subscales of somatic complaints, head pain complaints, cognitive complaints and thought dysfunction show.

Prior studies identified exercise and physical fitness as a public health resource (Gerber and Pühse, 2009) and exercise is recommended to maintain physical health (Garber et al., 2011), but the tendency toward complaining about physical and cognitive impairments (existing or non-existing) is also part of the personality and can be displayed with the described MMPI scales. The presented studies in our introduction about personality aspects in athletes and especially in (ultra-) marathon runners did not include items with regard to physical and cognitive complaints (Goldberg, 1993; McLeavey et al., 1984; Rauch et al., 1988; Egloff and Jan Gruhn, 1996; Hughes et al., 2003; Hashimoto et al., 2006; Freund et al., 2013).

Possible underlying mechanisms for our observation could be differences in pain perception: Previous studies found higher pain tolerance in endurance athletes [long-distance runners and triathletes, *N* = 86 (Egloff and Jan Gruhn, 1996), ultra-marathon runners, *N* = 11 (Freund et al., 2013)]. They argued that the personality trait of extraversion (increased in ultra-/marathon runners) could be associated with lower total resting levels of cortical arousals (Egloff and Jan Gruhn, 1996). As a result, higher levels of sensory stimulation are needed to induce positive/negative (e.g., pain perception) hedonic tone in extraverts. So the pain tolerance seems to be higher in extroverts due to this arousal deficit (Egloff and Jan Gruhn, 1996; Eysenck, 1967; Eysenck et al., 1982). It is still discussed whether the higher (physical) pain tolerance in endurance athletes like ultra-marathon runners is cause or consequence of the training (Freund et al., 2013).

Not only somatic, but also cognitive complaints were lower in our marathon runners compared to the non-active group.

This could be explained with improvements of cognitive function after endurance training due to, e.g., increased neuronal plasticity and better central vascularization (Voss et al., 2013). Moreover, other studies found an association of improved cognitive functioning after exercise in healthy adults (Gomez-Pinilla and Hillman, 2013; Kujach et al., 2018). Another possible explanation could be based on similar observations to the lower pain perception in marathon runners: the lower resting levels of cortical arousal could also lead to a lower sensation of not only somatic, but also cognitive deficits.

The lower results of *aberrant experiences* (ideas of persecution) point to a less distrustful personality of marathon runners.

When applying this finding to the personality traits in the big-five model, it would represent lower scores of openness, extraversion, conscientiousness, agreeableness and higher scores of neuroticism in the sedentary control group (Shi et al., 2018). Prior research indicated similar results (Hughes et al., 2003; Hashimoto et al., 2006; Freund et al., 2013; Roebuck et al., 2018). Again, this specific outcome was not measured in prior personality studies in athletes or specifically in (ultra-) marathon runners. A Finnish study included the Cynical Distrust Scale in a population-based study and found lower scores of distrust in the exercising group (frequency at least two or three times a week) (Hassmén et al., 2000) supporting our findings of less distrustful personalities in marathon runners. Less distrustful personalities are more open and experience-seeking and with that our results help clarify the conflicting findings of prior studies with ultra-marathon runners: they support two prior studies with similar findings of openness (Rauch et al., 1988; Hughes et al., 2003) and contradict other studies that found ultra-marathon runners to be more introverted (Hashimoto et al., 2006; Freund et al., 2013; Roebuck et al., 2018).

Our marathon runners exhibited lower scores of *demoralization, helplessness/hopelessness and stress/worry* compared to non-active controls. Prior studies described non-active groups as less self-motivated (Rauch et al., 1988; Hughes et al., 2003), pointing in the same direction without having examined the same scales. Most prior studies about personality traits used a variety of different questionnaires (often NEO-FFI or other questionnaires based on the “Big Five,” (Hughes et al., 2003; Hashimoto et al., 2006; Nikolaidis et al., 2018; Roebuck et al., 2018), so comparability to these studies is limited.

Interestingly, demoralization/helplessness/hopelessness and stress/worry are – beside others, including, e.g., concentration deficits – part of diagnostic criteria of depressive disorders and can coincide with somatic complaints (Kapfhammer, 2006). Regarding personality traits, these factors also contribute to the depressive personality disorder (or accentuation) (Vachon et al., 2009).

When applying these findings to clinical scenarios with psychiatric diagnoses, all three superordinate scales could be summarized with less depressive symptoms or less depressive personality traits in the broadest sense. In clinical practice, psychotic and depressive patients tend to score higher in neuroticism scales and lower in extraversion (Berenbaum and Fujita, 1994; Lewis et al., 2014) and therefore could exhibit similar personality traits.

To further analyze the possible depressive components and differentiate between personality traits and possible depressive disorders, we included the BDI and HAMD scales in our study. The self-rating BDI showed significantly higher scores in the sedentary controls compared to the marathon group (but still with values below the diagnostic cut-off for a manifest mild depressive disorder). The observer-rating scale HAMD on the other hand displayed no significant differences. This discrepancy between reported BDI and observed HAMD ratings has been linked to specific personality traits like high neuroticism and low extraversion in the big five model (Schneibel et al., 2012). This observation again supports earlier findings with higher scores of extraversion in endurance athletes and it points toward depressive personality traits more than objectifiable depressive symptoms in the meaning of depressive disorders (with no difference in HAMD scores between groups and overall values below diagnostic cut-offs in both scales).

We can conclude that we found traits of depressive personalities, but no depressive disorder, in the non-active group compared to the marathon runners.

In a second step we wanted to further analyze differences within the marathon runners (low versus high performer according to median IPAQ score) in order to possibly pronounce these findings. The “high performer” showed higher levels of hypomanic activation, cynicism, juvenile conduct problems, aggression, disaffiliativeness, social avoidance, disconstrained and lower levels of gastrointestinal complaints. Taken together, these traits point to a higher arousal in the high performer group and partly support the findings of prior studies of higher scores of vigor in marathon runners (Nikolaidis et al., 2018). All other scores, including the above described depressive personality traits were similar in both performance groups. This leads to the conclusion that there is a smooth transition between sedentary individuals with a (sub-)depressive personality, “regular runners” in between and hypomanic traits/increased psychological tension in committed runners.

### Influence of Sex and Age

As described, the marathon cohorts of the last years consist of more female and aged runners compared to those studies conducted a few decades ago (Nikolaidis et al., 2018), so we performed additional analyses regarding the impact of gender and age on personality traits. Results have to be interpreted cautiously, as the included female runners were significantly younger than the included male runners. We found that the female runners had higher scores of gastrointestinal complaints, demoralization, ideas of persecution, inefficacy, substance abuse, disaffiliativeness and mechanical-physical interests. Prior studies of non-athletes identified remarkably high prevalence rates of gastric and intestinal complaints in women (Avramidou et al., 2018) and gender-differences were established in regard to depressive symptoms being more pronounced in female cohorts (with our higher results of demoralization, disaffiliativeness and inefficacy pointing in the same direction) (Salk et al., 2017). The higher scores of mechanical-physical interests and drug abuse as two personality items of MMPI were more unexpected. Usually, prevalence of drug abuse is higher in men (McHugh et al., 2018). Thus, it may be speculated (similar with mechanical-physical interests) that the here displayed higher scores in these MMPI items can either point to female marathon runners exhibiting some “male” attributes or that these differences are related to the younger mean age of women in our group. Especially, mechanical-physical interests overlap with the age-comparison (with higher scores in the younger group and in the female group with also a younger mean age), so this sex-difference can be explained more likely by age-differences.

In the age-comparison we further identified lower scores of thought dysfunction, behavioral/externalizing dysfunction, demoralization, aberrant experiences, hypomanic activation, cognitive complaints, self-doubt, anxiety, family problem, disaffiliativeness, mechanical-physical interests, disconstrained, introversion/low positive emotionality in the older cohort of marathon runners. Vice versa, the lower percentage of female participants in this cohort has to be taken into account for interpretation especially in the overlapping scales (disaffiliativeness, demoralization, mechanical-physical interests). Other than that, the older marathon cohort seems to have lower scores in the sub-depressive scales (thought dysfunction, self-doubt, anxiety, cognitive complaints) and as well in the vigor-scales (hypomanic activation, behavioral/externalizing dysfunction, family problems and disconstrained). Comparing these results of less sub-depressive symptoms in the male and in the older marathon cohort to one of our main findings (less sub-depressive characteristics in the marathon runners compared to sedentary controls), it may be speculated that the impact of the older runners overweighs the higher proportion of female runners in the younger group. As the composition of marathon groups is still changing, the impact of age and gender on personality analyses in marathon runners need to be explored in more detail in future studies.

As limitation, one should be aware that dichotomizing continuous variables using median split may result in the loss of information especially regarding individual differences. Thus, our analyses should be interpreted with caution (MacCallum et al., 2002).

## Conclusion

This is the first study to evaluate personality traits in marathon runners with different performance groups and compare them to age and gender-matched sedentary controls using the newly developed MMPI-2-RF. We found that marathon runners experience less symptoms of somatic and cognitive complaints, less aberrant experiences and less hopelessness. We confirmed personality traits rather than objective depressive symptoms or disorders as underlying etiology. More committed runners did not present more pronounced traits, but higher levels of arousal. In our cohort that included more older runners and females compared to cohorts investigated some decades ago, the impact of these demographic variables explained in parts our personality findings. Future studies should further analyze the two-way interaction of depressive personality traits and endurance training to discriminate cause and effect.

## Data Availability Statement

The datasets generated for this study are available on request to the corresponding author.

## Ethics Statement

The studies involving human participants were reviewed and approved by the ethics committees of both the Ludwig-Maximilians University Munich Ethics committee (approval reference number 17-148) and the Technical University Munich (approval reference number 218/17 S). The patients/participants provided their written informed consent to participate in this study.

## Author Contributions

AH, JoS, PF, ML, and AR conceptualized the study. AR and RE wrote the first draft of the manuscript. IP, BP, and JuS collected data. AR, AH, and RE made the statistical analyses. All authors revised the manuscript and approved of the final version. All authors agreed to be accountable for all aspects of the work in ensuring that questions related to the accuracy or integrity of any part of the work are appropriately investigated and resolved.

## Conflict of Interest

The authors declare that the research was conducted in the absence of any commercial or financial relationships that could be construed as a potential conflict of interest.

## Footnotes

1 http://apps.who.int/trialsearch/
